# Hand Extension Robot Orthosis (HERO) Grip Glove: enabling independence amongst persons with severe hand impairments after stroke

**DOI:** 10.1186/s12984-020-00659-5

**Published:** 2020-02-26

**Authors:** Aaron Yurkewich, Illya J. Kozak, Debbie Hebert, Rosalie H. Wang, Alex Mihailidis

**Affiliations:** 1 Institute of Biomaterials and Biomedical Engineering, University of Toronto, Toronto, Canada; 2 University Health Network - Toronto Rehabilitation Institute – KITE, Toronto, Canada; 3 Bioengineering, Imperial College London, London, UK; 4 Occupational Science and Occupational Therapy, University of Toronto, Toronto, Canada

**Keywords:** Portable robotic glove, Exoskeleton, Assistive technology, Stroke, Hand, Rehabilitation, Activities of daily living

## Abstract

**Background:**

The Hand Extension Robot Orthosis (HERO) Grip Glove was iteratively designed to meet requests from therapists and persons after a stroke who have severe hand impairment to create a device that extends all five fingers, enhances grip strength and is portable, lightweight, easy to put on, comfortable and affordable.

**Methods:**

Eleven persons who have minimal or no active finger extension (Chedoke McMaster Stage of Hand 1–4) post-stroke were recruited to evaluate how well they could perform activities of daily living and finger function assessments with and without wearing the HERO Grip Glove.

**Results:**

The 11 participants showed statistically significant improvements (*p* < 0.01), while wearing the HERO Grip Glove, in the water bottle grasp and manipulation task (increase of 2.3 points, SD 1.2, scored using the Chedoke Hand and Arm Inventory scale from 1 to 7) and in index finger extension (increase of 147^o^, SD 44) and range of motion (increase of 145^o^, SD 36). The HERO Grip Glove provided 12.7 N (SD 8.9 N) of grip force and 11.0 N (SD 4.8) of pinch force to their affected hands, which enabled those without grip strength to grasp and manipulate blocks, a fork and a water bottle, as well as write with a pen. The participants were ‘*more or less satisfied’* with the HERO Grip Glove as an assistive device (average of 3.3 out of 5 on the Quebec User Evaluation of Satisfaction with Assistive Technology 2.0 Scale). The highest satisfaction scores were given for safety and security (4.6) and ease of use (3.8) and the lowest satisfaction scores were given for ease of donning (2.3), which required under 5 min with assistance. The most common requests were for greater grip strength and a smaller glove size for small hands.

**Conclusions:**

The HERO Grip Glove is a safe and effective tool for enabling persons with a stroke that have severe hand impairment to incorporate their affected hand into activities of daily living, which may motivate greater use of the affected upper extremity in daily life to stimulate neuromuscular recovery.

## Background

Fifteen million individuals worldwide experience a stroke each year with 50,000 of these cases occurring in Canada [1]. Approximately two-thirds of these individuals will experience neurological deficit [2] and half will never fully recover the hand function required to perform activities of daily living independently [3]. Stroke survivors with severe hand impairment have difficulty producing hand motion and grip force and their increased muscle tone, spasticity and contractures keep their hand clenched in a fist. These stroke survivors have the potential to attain functional improvements years after their stroke by constantly incorporating the affected hand into activities of daily living (ADLs) and additional goal-directed tasks during their therapy exercises and daily routines [4–6].

There are many barriers to incorporating the affected hand into exercises and daily routines including time, discomfort, safety risks and mental and physical effort. Personalized, high-intensity, coaching and motion assistance is required to overcome these barriers but is often inaccessible to stroke survivors. The time and resource commitments are too substantial for many clinics to supply at a sufficient intensity and additional rehabilitation technologies and services can be inaccessible due to high cost, location and availability [7, 8]. As a result, stroke survivors often do not regain the hand range of motion (ROM), strength and coordination required to perform ADLs independently. Affordable and accessible rehabilitation technologies and services that enable stroke survivors with severe hand impairment to incorporate their affected hand into ADLs are needed to maximize neuromuscular recovery and daily independence.

### Design targets for wearable hand robots

A main goal for wearable hand robots is to provide the hand function assistance and rehabilitation required to enable people after stroke to perform ADLs independently. Able-bodied individuals move their fingers through a ROM of 164^o^ during activities of daily living, as calculated by summing the differences between the extension and flexion joint angles of the distal interphalangeal (DIP), proximal interphalangeal (PIP) and metacarpophalangeal (MCP) joints [9]. The thumb moves through a ROM of 40^o^, as calculated by summing the differences between the extension and flexion joint angles of the thumb’s interphalangeal (IP) and MCP joints [9]. Grip forces averaging 67 N are exerted [10] and a combination of hand postures are used (i.e. a tripod pinch was used during 38% of the activities of daily living evaluated, extended hand (13%), cylindrical grasp (12%), lumbrical grasp (10%), lateral pinch (9%)) [11].

### Capabilities of wearable hand robots

Wearable hand robots have manipulated able-bodied participants’ relaxed hands to provide 129^o^ of index finger ROM, 83 N of grip strength as measured using a hand dynamometer, and 7 hand postures in Rose et al. [10]. However, when these robots are evaluated with impaired hands the assistive capabilities have been much lower. For studies by Cappello et al. and Soekadar et al. with six and nine persons with impaired hands following a spinal cord injury, wearable hand robots have increased grip strength to 4 N [12] and ADL performance to 5.5 out of 7 on the Toronto Rehabilitation Institute - Hand Function Test by assisting pinch and palmar grasp postures [12, 13]. For a study by Yurkewich et al. with five persons with severely impaired hands following stroke (no voluntary index finger extension), a previous version of the HERO Grip Glove named the HERO Glove increased ROM to 79^o^ and improved water bottle and block grasping performance [14]. Refer to [14] for a supplementary table detailing recently developed wearable hand robots, their capabilities and their evaluation results. Hand robots need to be improved to generate strong extension and grip forces that overcome muscle tone and securely stabilize various object geometries, such as a water bottle and a fork. These robots should also be easy to put on clenched hands, comfortable during multiple hours of use, lightweight so as not to affect the motion of weak arms and affordable so they are accessible to people with limited income even though these considerations create design tradeoffs that sacrifice assistive capabilities [14, 15].

A number of sensor types (i.e. button [12, 14, 16], electromyography [17, 18], motion [10, 14], force [19], voice [20], vision [21, 22] and electroencephalography [13] have been selected to control robot assistance based on varied motivations such as robust operation or motivating neuromuscular activation. However, other than button control, these control strategies are still in an experimental stage that requires experts to manually tune each user’s orthosis [17].

A single study evaluating two stroke survivors’ satisfaction with a wearable hand robot was completed by Yap et al. [16] to understand their needs and preferences in wearable hand robot design. More rigorous studies would further inform designers on how to adapt their wearable hand robots to maximize the intended users’ satisfaction and arm and hand use.

**Fig. 1:**
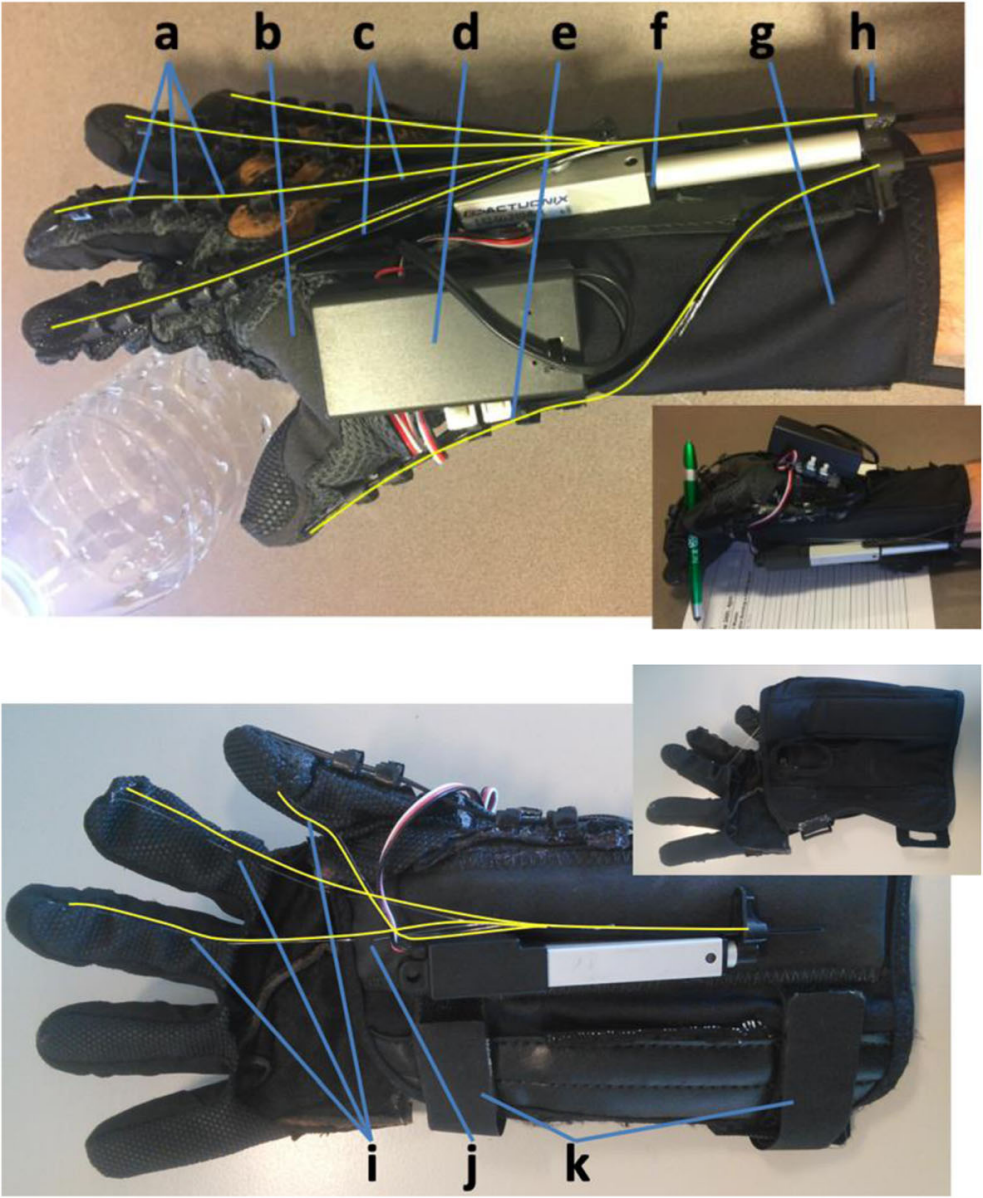
The HERO Grip Glove assists finger and thumb extension and flexion to enable users to grasp large and small objects. The HERO Grip Glove consists of (**a**) cable tie guides, (**b**) an open-palm glove, (**c**) cable tie tendons for extension, (**d**) a 9 V battery case with the battery inside and the microcontroller with an inertial measurement unit mounted between the case and the glove, (**e**) buttons to control the manual mode and select between the manual and automatic modes used in [14], (**f**) a linear actuator, (**g**) a foldable wrist brace, (**h**) cable tie pawls for pre-tensioning, (**i**) fishing wire tendons for flexion, (**j**) tendon anchor points on the wrist brace and (**k**) Velcro straps to secure the glove. The glove folds open to ease donning. The dorsal and palmar tendons’ routing paths are highlighted in yellow

This article presents the portable Hand Extension Robot Orthosis (HERO) Grip Glove, including its novel design features and the evaluation of its assistive capabilities and usability with 11 stroke survivors with severe hand impairments. The HERO Grip Glove, shown in Fig. 1, assists five-finger extension, thumb abduction and tripod pinch grasping using particular cable materials and routing patterns and only two linear actuators. A fold-over wrist brace is used to mount the electronic components, support the wrist, and ease donning. The robot is controlled by hand motion or a button. The robot is open source for broad access, untethered and lightweight for unencumbered use throughout daily routines, and soft to conform to hands and objects of varying geometries. The HERO Grip Glove increases range of motion and ADL performance with large and small objects and increases grip strength for those without grip strength. The participants’ quantitative and qualitative feedback from their user satisfaction questionnaires provides guidance for assistive technology developers and motivation for deploying the HERO Grip Glove to stroke survivors for use throughout their daily routines.

## Methods

### HERO Grip Glove design

The HERO Grip Glove, shown in Fig. 1, provides finger extension assistance and thumb extension and abduction assistance by extending the actuator on the dorsal side of the wrist to apply tension along the dorsal tendons and retracting the actuator on the palmar side of the wrist to release the tension along the palmar tendons. The HERO Grip Glove provides index and middle finger flexion assistance and thumb opposition assistance by extending the actuator on the palmar side of the wrist and retracting the actuator on the dorsal side of the wrist.

The HERO Grip Glove was iteratively designed with stroke survivors and therapists to meet their design specifications, shown in Table 1. These design specifications are shown in Table 1 of [14], and are based on qualitative and quantitative feedback gathered from stroke survivors and therapists. The HERO Grip Glove has additional components and capabilities that were not present in the HERO Glove [14], including a palmar actuator and flexion tendons for grip assistance, dorsal tendons for ring and little finger extension, and a wrist brace that mounts the actuators more rigidly and keeps the wrist in a neutral position.

**Table 1:** Therapist and stroke survivor design specifications and the HERO Grip Glove’s capabilities

Design Specification	HERO Grip Glove Capability
Apply at least 10 N of extension to each finger and the thumb to enable cylindrical grasping	TBD, Applies 80 N of tension force across artificial tendons to assist extension of all fingers and the thumb. The force exerted on the fingers was not measured.
Apply at least 15 N of grip force and ideally 67 N	X*,* Supplies 12.7 N (SD 8.9 N) of grip force and 11.0 N (SD 4.8) of pinch force through the index and middle fingers and thumb. Larger forces can be applied if the user volitionally generates force to supplement the glove’s force.
Untethered, weighs less than 400 g, and profile less than 5 cm to avoid obstructing ADL performance	✔*,* Wireless, weighs 284 g (0.63 lb) in fully-operable state (with all components in Fig. 1 included), the maximum profile protrusion is 2 cm (battery case height).
Controlled by the affected hand with less than a 16% false positive rate	~, The IMU (affected hand) control mode showed a 90% grasp intent detection accuracy (10% false positive rate) during the BBT. Additional instruction was required to complete the water bottle task. Manual and automated modes were rated 3.8 and 3.4 (out of 5) for ease of use during an ADL, respectively.
Does not hyperextend affected finger joints and pressure points eliminated	TBD*,* No hyperextension was observed and no complaints about pain were mentioned. The pressure exerted on the fingers was not measured and the glove could obstruct observation of joint hyperextension.
Device cost less than 500 USD	✔*,* Components cost 300 USD, device available at cost through MakersMakingChange.com
Able to don on flexed and flaccid hands in under 5 min, ideally without assistance	~*,* Each user donned the glove onto their spastic (flexed) or flaccid hand in under 5 min. The glove was doffed in less than 1 min. Assistance was needed for donning but not doffing.
Enables stroke survivors with severe hand impairment to extend their fingers	✔*,* Increased finger extension (147^o^, SD 44) and range of motion (145^o^, SD 38) for each stroke survivor.
Enables stroke survivors to use their affected hand in activities of daily living	✔*,* Enabled each participant to successfully grasp and release blocks during the BBT and to grasp, lift, manipulate and release a water bottle, a fork and a pen. The glove’s effectiveness was rated 3.4 (out of 5).
Additional HERO Grip Glove Specifications	
Length x Width	33 cm × 10 cm
Safety Features	Screw-drive transmission to limit extension, Velcro wrist strap for doffing in less than 1 min
Actuated ROM	Approximately 158^o^ for relaxed stroke hands (summed MCP-PIP-DIP bending)
Speed of Actuation	Approximately 21^o^/second (summed MCP-PIP-DIP bending)
Battery Life	> 4 h of continuous use

*The **✔** denotes “Meets specification”, **~** denotes “Partially meets specification”, TBD denotes “To Be Determined through further studies” and **X** denotes “Does not meet specification”

#### Grip and pinch strength assistance

Hand robots with artificial flexor tendons routed through the palm have demonstrated large grip forces [10, 21]; however, open-palm designs are much easier to put onto clenched fingers [14, 16, 23]. We found that these flexor tendons could be moved out of the way during donning if the tendons were routed through the palmar side of a folding wrist brace (epX Wrist Control, Medium), as shown in Fig. 1. The wrist brace is secured to the user by tightening the Velcro palm and forearm straps. The wrist brace provides improved comfort, safety and wrist posture and a rigid mounting location for the actuators (Actuonix, L12-R, 210:1, 80 N max force, 50 mm stroke length) and electronics (tinyTILE Intel Curie microcontroller, 9 V Energizer Lithium battery). The metal palmar support inside the wrist brace can be removed if the user prefers a more flexible wrist.

The flexor tendon routing paths, shown in Fig. 1, were specifically chosen to provide a tripod pinch and increase grip strength, grasp workspace, comfort, sensation and ease of donning. The flexor tendons were anchored approximately 5 mm distal to the thumb IP joint and the index and middle finger DIP joints using four backstitches and a knot. Two tendons were added at the thumb to balance the force from both finger tendons. The tendons were routed inside the glove at the phalanges and external to the glove at the PIP and MCP joints. To further ease finger donning for clenched hands, the tendons were positioned on the radial side of the thumb and fingers and tendons were not added for the ring and little fingers. For hands that are initially extended, tendons could be added on the ulnar side of the fingers and on the ring and little fingers without making donning much more difficult. The thumb and finger tendons route through the wrist brace on top of the thenar muscles, which creates the finger flexion, thumb opposition and hand curvature required for the thumb to touch the index and middle fingertips. Each tendon was attached to the same actuator to keep the glove lightweight and affordable. The fingertip force generated by each finger and thumb tendon is shown in Eq. 1. The theoretical tip pinch and cylindrical grip forces are 16 N and 32 N for a male’s hand using an 80 N actuator force. The under-actuated tendon system allows the joints to self- align to the object’s shape. The flexibility of the glove material (Mechanix, Men’s Large) and fishing wire tendons (Stren, 14 lb) allow large and small objects to be gripped with the same actuator stroke length. The grasp workspace is slightly smaller than that of an able-bodied hand because the flexor tendons protrude approximately 1 cm from the MCP joint under tension.

1 $$ Ft\ast Dtj/ Dpo= Fo $$

where Ft is the tension force on the tendon, Dtj is the normal distance between the tendon and the finger joint axis, Dpo is the distance from the center of the palm to the center of pressure on the object and Fo is the tendon’s contribution to the grip force on the object. For the HERO Grip Glove, the approximate values for an index finger tendon’s contribution to a pinch grip are Ft = 80 N, Dtj = 20 mm, Dpo = 100 mm, Fo = 16 N.

#### Finger and thumb extension and abduction assistance

The HERO Grip Glove’s extension mechanism is an improvement over its previous version, which required stronger extension force, five-finger extension and thumb abduction to meet user-defined specifications [14]. A greater extension force was achieved by rigidly mounting the actuator to the wrist brace, restricting migration to less than 1 cm when applying strong forces. The pressure exerted by the extension mechanism is well-distributed by the gloves padded fingers and the wrist brace’s large surface. The stronger extension force allows the glove to extend all five fingers using a single actuator, without sacrificing the extension motion of any one finger. The cable ties used for the extensor tendons were bolted to a single central tendon, which helped the fingers abduct from each other. The single tendon was mounted to the actuator using an adjustable cable tie pawl so the maximum finger extension could be adjusted quickly based on the user’s finger lengths. Additionally, the flexor tendons’ tension increases near full extension, which blocks hyperextension. The thumb cable tie was routed through an additional cable tie guide positioned above the abductor policis brevis to increase thumb abduction and provide a functional grasp preparation posture. The right and left-handed HERO Grip Gloves were created from the anatomical measurements of an able-bodied male whose hands fit Medium and Large-sized (United States) gloves (hand length: 200 mm, palm breadth: 90 mm). Adult hand sizes generally range between small and extra-large glove sizing standards and some people after stroke have additional hand swelling [24, 25]. A Large-sized glove was chosen as a compromise between different sized hands, so each participant could be tested with the same sized glove without the glove being too loose.

#### Manual and automatic control options

Two control modes were used to operate the HERO Grip Glove, which are unmodified from its predecessor [14]. The control diagram is shown in Supplementary Figure 1 of [14]. In the manual mode, the more proximally located button of the two buttons shown in Fig. 1 can be pressed by the user’s unaffected hand or by the therapist to toggle between extension and flexion assistance. The more distal button can be pressed to toggle between manual and automatic control. In the automatic mode, the robot switches from extension assistance to grip assistance, and vice versa, each time the hand is moved quickly. Specifically, the robot’s assistance switches once the hand is moved at a rotational absolute velocity that exceeds 23^o^/s and then slows to less than 23^o^/s for at least 0.8 s. The hand’s motion is sensed by the inertial measurement unit (IMU) gyroscope that is mounted to the glove on the dorsal surface of the hand. This control strategy was selected because the hand motions produced during pick and place tasks intuitively trigger the extension and grip assistance and five people after stroke used this control strategy effectively during previous Box and Block Tests [14].

### Participant recruitment

Observational case studies with stroke participants with limited active finger extension were completed to evaluate the HERO Grip Glove’s usability and efficacy in increasing finger extension angle, finger range of motion, grip and pinch strength and ADL performance. A convenience sample of stroke survivors was recruited by therapist referral for outpatient participants and the Toronto Rehabilitation Institute–University Health Network (TRI-UHN) central recruitment process for inpatient participants. This study was approved by the UHN Institutional Review Board #16–6198 and each participant provided informed consent to participate in the study. The authors administered the study methods for all stroke survivors, after being trained by an occupational therapist. Outpatients did not receive additional therapy on the day of the study. Inpatients completed scheduled therapy sessions on the same day as the study.

#### Inclusion criteria

*•* Stroke survivors more than 1 week post-stroke.

• Chedoke-McMaster Stroke Assessment Stage of the Hand (CMSA-Hand) [26] between 1 and 4, inclusive (moderate to severe hand impairment).

### Assessments

#### Range of motion, tone and spasticity assessments

The stroke participants were seated with their affected hand and arm resting on a table at approximately elbow height. The researcher measured the bend angle of the index finger MCP, PIP, and DIP joints using a dorsal finger goniometer (JAMAR, analog, 5^o^ resolution) in four positions, passive extension, active flexion, active extension and then passive flexion, as in [14]. Further figures, term definitions, and details for these ROM measurements and calculations are provided in Supplementary Figure 1 and in the Supplementary Materials of [14]. The accuracy of finger joint angle measurement using a finger goniometer is within the device’s resolution (approximately 3^o^) [27, 28]. All of the goniometer measurements were performed by the same researcher. The researcher was prepared to stop applying force if the participant felt moderate pain (i.e. pain rating above 3 out of 10 on the Numeric Pain Rating Scale [29]), but this level of pain was never reported during the study. The finger joints were not extended past straight to avoid potential injury, so the maximum extension was 0^o^ for each joint. Tone and spasticity in the index finger was assessed using the Modified Modified Ashworth Scale (MMAS) [30] and Modified Tardieu Scale (MTS) [31].

The robot-assisted (R-A) ROM was measured using the same instruments, arm posture and finger joints as in the unassisted ROM measurements. The glove was donned with assistance to ensure proper alignment and the robot extended the fingers to ensure safe operation. The finger extension cable ties were adjusted to provide maximal finger extension and then the flexion tendons were tightened to provide maximal grip strength without limiting extension. The participants were asked to keep their hand relaxed as the robot assisted their motion to isolate the robot’s effect on ROM. The researcher positioned the finger goniometer on the dorsal side of the index finger, beside the artificial tendon. The researcher palpated the finger joints and phalanges to ensure the finger goniometer was properly aligned and flush against the glove and the glove was flush against the skin. The finger’s outline can be seen against the dorsal side of the glove. The robot’s assistance kept the finger stationary during the measurements. The researcher measured the index finger MCP, PIP and DIP joint angles in R-A flexion and then R-A extension. The R-A ROM was calculated by subtracting the R-A extension joint angles from the R-A flexion joint angles.

Participants were then asked to flex their hand to supplement the glove’s flexion assistance, to explore how much of an effect volitional hand muscle activation would have on robot-assisted ROM. This assessment was added after P3, 4, 6, 8 and 11 had completed the study and these measurements were not used in the tables or statistical analyses.

#### Grip and pinch strength assessments

The participants’ grip strength and tripod pinch strength were measured using a dynamometer (JAMAR, analog, Sammons Preston, Model 5030 J1, 5 lbs. gradations, estimated resolution to 1 lb. (4.4 N)) and pinch gauge (JAMAR, analog, Sammons Preston, Model 749,805, gradations and resolution of 1 lb. (4.4 N)). The grip and tripod pinch measurements were repeated three times and the average values are reported. The participants’ fingers were positioned around each gauge with the arm resting on the table. For the tripod pinch measurement, the thumb was positioned on top of the pinch gauge’s force pad and the index and middle fingertips were positioned underneath. The researcher supported the gauge and asked the participant to grip and pinch with their maximum strength.

Robot-assisted grip and pinch strength were measured while the participants were asked to keep their hand relaxed to isolate the robot’s effect on grip and pinch force. Robot-assisted strength measurements were added to the study after P4 and P11, chronologically the first two participants in the study. P9 did not complete the grip strength assessment because he felt discomfort where the stitch anchoring the thumb tendon to the glove pressed on the thumb tip and metal dynamometer handle. The grip dynamometer malfunctioned during P2’s trial.

Participants were then asked to flex their hand to supplement the glove’s pinch force assistance, to explore how much of an effect volitional hand muscle activation would have on robot-assisted pinch force. This assessment was added after P3, 4, 6, 8 and 11 had completed the study and included only participants that could generate pinch force without assistance. These measurements were not used in the tables or statistical analyses.

#### Box and block test assessment

The Box and Block Test (BBT) is a test of participants’ capability to grasp individual 2 cm × 2 cm wooden blocks from within a wooden box with 150 blocks, lift them across a 15.2 cm barrier at their midline, and release the blocks, in 1 min [32]. On average, able-bodied subjects over 75 years of age can transfer more than 60 blocks [33]. This test has been used to evaluate previous robotic hand orthoses [14, 34]. Participants who are able to perform the BBT may also be able to perform daily tasks with similar sized items, such as utensils, toothbrushes and handles. Participants were asked to perform this task without robot assistance and with the HERO Grip Glove in both the manual and automatic mode. Participants were given up to 5 min to practice the task unassisted and up to 5 min to practice the task robot-assisted before being evaluated.

The BBT and the Water Bottle Task, Fork Task and Pen Task explained below, were modified in that forearm support was provided manually by the researcher or the unaffected side if this assistance was needed to perform the task.

#### Water bottle task assessment

The water bottle grasp task, an ADL, was assessed using the Chedoke Arm and Hand Activity Inventory (CAHAI) scale from 1 (unable to perform task) to 7 (able to perform the task independently and quickly without assistance from the unaffected hand) [35]. Participants were seated with their hand resting on a table and a water bottle placed approximately 20 cm in front of their torso. Participants were instructed to reach with their affected arm to grasp the water bottle, lift the water bottle and hold the water bottle while twisting off the lid with the opposite hand. The participants attempted the water bottle task without wearing the HERO Grip Glove and then reattempted the task while wearing the HERO Grip Glove and using the manual control mode. The automatic mode was not used because the participants in [14] found it challenging to lift the arm and twist off the lid without generating high arm accelerations that triggered a false-positive hand extension. An empty plastic water bottle was used as opposed to the coffee jar recommended for the CAHAI, because it was safer to drop, easily accessible, of comparable diameter (76 mm), and light enough to lift with a weak but active arm.

#### Fork task and pen task assessments of grasp and manipulation capability

The participants were asked to use only their affected hand to pick up a fork from the table, lift the fork and manipulate it in the air. They were assessed using the Toronto Rehabilitation Institute Hand Function Test (TRI-HFT) scale from 1 (unable to grasp the object) to 7 (able to grasp and lift the object completely off the supporting surface and manipulate the object using an active grasp with normal function). Participants that were unable to pick the object off the table reattempted the task with assistance from the unaffected hand to place the object in the affected hand, as in [12].

The participants reattempted the fork task while wearing the HERO Grip Glove using the manual control mode and were assessed using the TRI-HFT scale. The participants with affected dominant hands that were unable to manipulate the fork without the HERO Grip Glove’s assistance attempted to grasp, lift, manipulate and use a pen to write with the HERO Grip Glove. The participants were allowed to use the unaffected hand to place the object in the affected hand, if needed.

#### Usability testing – Quebec user evaluation of satisfaction with assistive technology version 2.0 (QUEST)

Participants were asked to evaluate how usable the HERO Grip Glove would be as an assistive device throughout their daily routine, using the QUEST scale (0 = not satisfied at all, 5 = very satisfied) [36]. The assessment was administered verbally and transcribed by the researchers due to the participants’ difficulties writing, after the above tasks were completed. This assessment was chosen because it is a reliable measure that provides direct quantitative and qualitative feedback as to which design specifications should be improved [37]. This assessment was added to the study after P4, chronologically the first participant in the study. Further data points were not available because the participants needed to leave for other commitments or thought a metric was irrelevant (e.g. rating durability after a single session) or difficult to score using a Likert scale. The video recordings of the trials were used to measure the time required for each participant to don the HERO Grip Glove at the beginning of the study and remove it at the end of the study.

### Statistical analysis

The Shapiro-Wilk test was used to evaluate if the finger extension, range of motion, grip strength, pinch strength, BBT, water bottle task and fork task datasets were normally distributed (α = 0.05). For the without glove versus with glove comparisons, a paired t-test was used to determine if the normally distributed datasets (i.e. finger extension, range of motion, grip strength and pinch strength) were statistically significant (α = 0.05) [38] and the Wilcoxon signed-rank test was used to determine if the non-normally distributed datasets (i.e. BBT, water bottle task and fork task) were statistically significant (α = 0.05) [12].

## Study results

### Participants

**Fig. 2:**
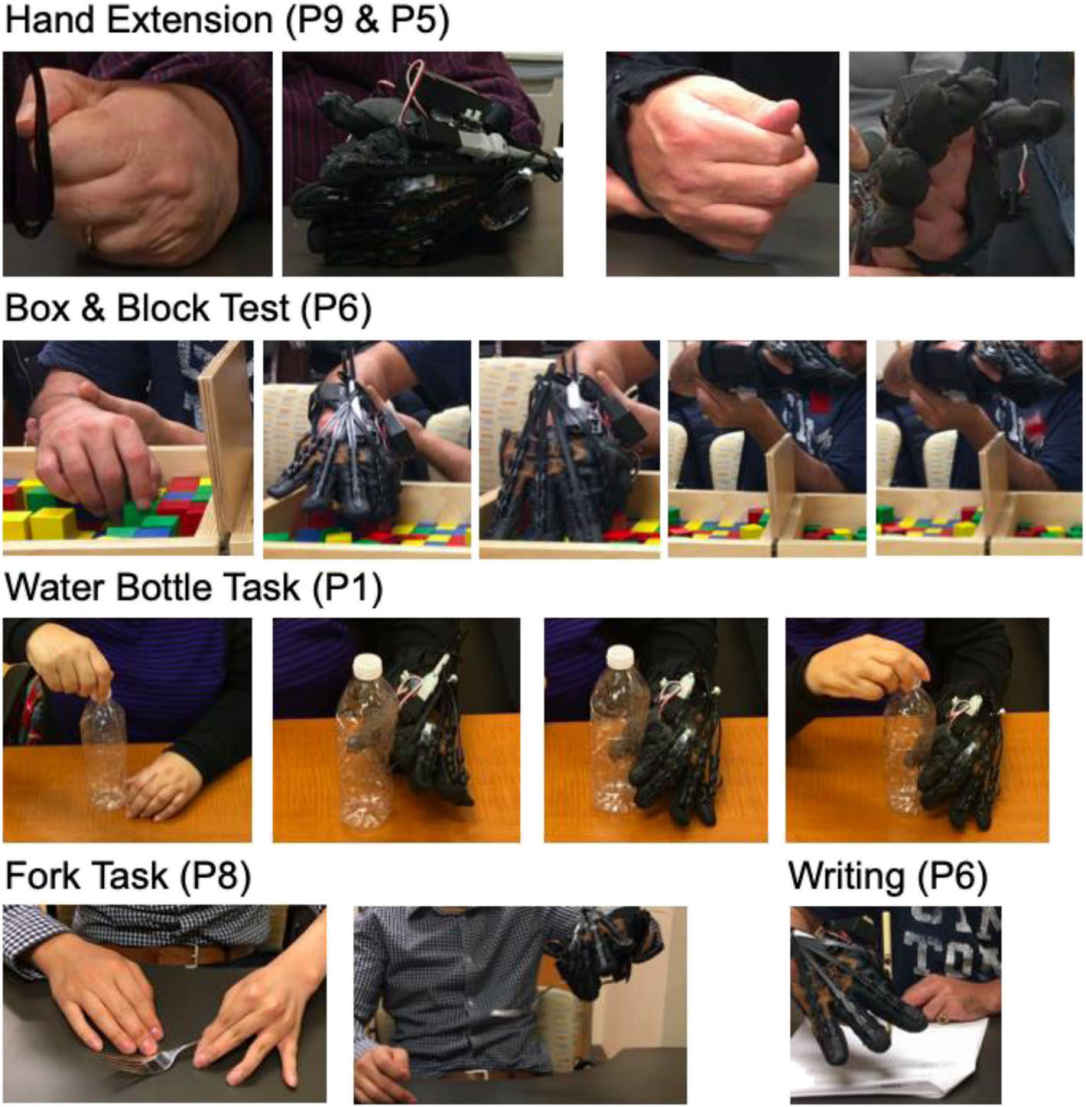
Comparative images taken during the study assessments of the affected hand without and with the HERO Grip Glove

This study involved 11 stroke survivors (3 acute, 8 chronic) with a broad range of severe hand impairments. The participants are numbered by ascending CMSA-Hand score to simplify the interpretation of the results, and their demographics are shown in Table 2. The study results for finger extension, ROM, grip and pinch strength, task performance and usability are shown in Tables 3, 4, 5, 6 and Fig. 2. The participants ranged from CMSA-Hand level 1 (flaccid paralysis) to 4 (able to fully extend and then flex the hand, but not able to flex and then extend the hand). Tone and spasticity (resistance to assisted finger extension) was measured using the MMAS and MTS and ranged from 0 (no increase in tone) to 2 (more marked increase in tone), with no score differences between the two measures. Four of 11 participants showed a reduced sense of touch in their fingers, palm and forearm, using the Fugl-Meyer Assessment – Sensation to Light Touch (FMA-S) [39]. Extra caution was taken to check for redness and marks on the skin in these cases. Four participants (P1, 3, 4, 10) began the study with mild pain (1 to 3 out of 10) and no participants reported an increase in pain during the study, as assessed using the Numeric Pain Rating Scale [29]. Three participants (P1, 2, 10) used a wheelchair and the other participants walked independently with a cane if needed. A few participants mentioned owning a resting hand splint but rarely using it and no participants arrived for the study with any upper extremity assistive devices.

**Table 2:** Stroke participant demographics and hand function

	Months Post Stroke	CMSA Hand	MMAS	FMA-S	Aff / Dom Hand	Gender	Age
P1	0	1	0	2	L/R	F	65
P2	2	1	1	1	R/R	M	48
P3	37	1	2	2	R/R	M	58
P4	31	3	0	2	L/R	M	68
P5	47	3	0	2	R/R	M	64
P6	42	3	0	2	R/R	M	44
P7	77	3	1	1	R/R	F	47
P8	208	3	2	1	L/R	M	34
P9	412	3	2	2	L/R	M	84
P10	2	3	2	1	R/R	M	32
P11	198	4	0	2	L/L	F	50

**Table 3:**
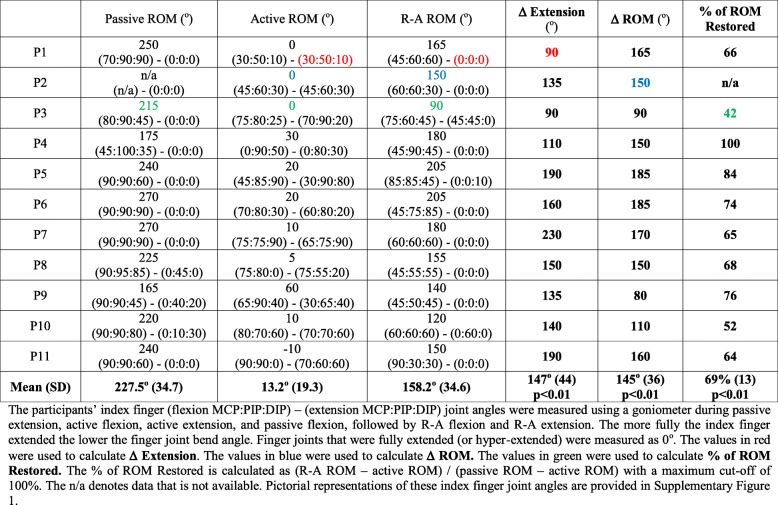
Index finger extension and range of motion (rom) assessments with and without robot assistance (R-A)

**Table 4:** Grip and pinch strength with and without robot assistance (R-A)

	Grip Strength (N)	R-A Grip Strength (N)	Δ Grip Strength (N)	Pinch Strength (N)	R-A Pinch Strength (N)	Δ Pinch Strength (N)
P1	0	22.2	**22.2**	0	10.4	**10.4**
P2	n/a	n/a	**n/a**	0	17.8	**17.8**
P3	0	8.9	**8.9**	20.8	17.8	**−3**
P4	3	n/a	**n/a**	3.7	n/a	**n/a**
P5	75.6	4.4	**− 71.2**	19.3	8.9	**−10.4**
P6	17.8	13.3	**−4.4**	17.8	4.4	**−13.3**
P7	28.2	4.4	**−23.7**	20.8	11.9	**−8.9**
P8	25.2	8.9	**−16.3**	30.4	11.1	**−19.3**
P9	86	n/a	**n/a**	32.6	4.4	**−28.2**
P10	50.4	26.7	**−23.7**	14.8	11.9	**−3**
P11	22.2	n/a	**n/a**	22.2	n/a	**n/a**
**Mean (SD)**	**30.8 (30.5)**	**12.7 (8.9)**	**−15.5 (29.9)**	**16.6 (11.1)**	**11.0 (4.8)**	**−6.4 (14.1)**

Average grip strength and pinch strength over three repeat trials measured using a dynamometer and pinch gauge

**Table 5:** Task-based assessments with and without robot assistance (R-A)

	Box & Block Test (BBT)	R-A BBT - Button Mode	R-A BBT - Auto Mode	Δ BBT	Water Bottle Task (WBT)	R-A WBT	Δ WBT	Fork Task	R-A Fork Task	Δ Fork Task
P1	0	2^b^	3^b^	**3**	1^b^	3^a^	**2**	1^c^	6^c^	**5**
P2	0	3^a^	4^a^	**4**	2^b^	4^a^	**2**	1^c^	6^c^	**5**
P3	0	3^a^	3^a^	**3**	2^b^	4	**2**	n/a	6^c^	**n/a**
P4	7^a^	4^a^	n/a	**−3**	n/a	3^b^	**n/a**	n/a	n/a	**n/a**
P5	0	1^a^	n/a	**1**	2	4^b^	**2**	n/a	n/a	**n/a**
P6	0	3^a^	3^a^	**3**	3	6	**3**	1^c^	6^c^	**5**
P7	2	4	4	**2**	4	4	**0**	6	6^c^	**0**
P8	0	3	3	**3**	2^b^	6	**4**	5^c^	6	**1**
P9	17	4	4	**−13**	2	4	**2**	6	6^c^	**0**
P10	0	2^a^	3^a^	**3**	2^b^	4^b^	**2**	1^c^	6^c^	**5**
P11	5	3	3	**−2**	2	6	**4**	n/a	n/a	**n/a**
**Mean (SD)**	**2.8 (5.3)**	**2.9 (0.9)**	**3.3 (0.5)**	**0.36 (5.0)**	**2.2 (0.8)**	**4.5 (1.1)**	**2.3 (1.2)** **p < 0.01**	**3.0 (2.5)**	**6.0 (0)**	**3.0 (2.5)** **p < 0.01**

Participants attempted to use their affeceted hand to grasp blocks, transfer them over a barrier and release them. The task was completed without wearing the glove and then while wearing the glove and operating it using the manual and automatic modes. ^a^ Signifies participants that supported their affected forearm with their unaffected hand. ^b^ Signifies participants whose affected forearm was supported by the researcher

Participants attempted to grasp and lift a water bottle with their affected hand and then hold the water bottle while twisting off the lid with their unaffected hand. This task was then repeated while wearing the HERO Glove, with its assistance controlled by the user using the physical button. Breakdown of the **CAHAI** scoring metric: 1 – not able to use affected hand, 2 – able to stabilize the object with the affected hand and complete the task with physical assistance, 3 - able to stabilize and manipulate the object with the affected arm and hand with physical assistance, 4 – all components completed with the affected hand with only light touch assistance, 5 – all components completed with only verbal cueing and help donning orthoses, 6 – all components completed without assistance, but with support from assistive devices, 7 – all components completed safely, quickly, and smoothly

Participants attempted to grasp and lift a fork from the table with their affected hand and then hold and manipulate the fork in the air. This task was then repeated while wearing the HERO Glove, with its assistance controlled by the user using the physical button. Breakdown of the **TRI-HFT** scoring metric: 1 - unable to grasp the object, 2 – able to grasp using a passive grasp but unable to lift the object successfully off the supporting surface, 3 – able to grasp using an active grasp but unable to lift the object successfully off the supporting surface, 4 – able to grasp and lift the object completely off the supporting surface using a passive grasp but unable to manipulate the object, 5 – able to grasp and lift the object completely off the supporting surface using an active grasp but unable to manipulate the object, 6 – able to grasp and lift the object completely off the supporting surface and manipulate the object using a passive grasp appropriately, 7 – able to grasp and lift the object completely off the supporting surface and manipulate the object using an active grasp with normal function. ^c^**The unaffected arm provided assistance to place the fork in the hand and/or support the arm during reaching, lifting and/or manipulation**

**Table 6:** HERO Grip Glove – Quebec User Evaluation WIth Assistive Technology (QUEST) Version 2.0

	Size	Weight	Ease of Donning	Safe & Secure	Durability	Ease of Use- Button Mode	^a^Ease of Use- Auto Mode	Comfort	Effective	Overall Average
P1	4	1	3.5	5	4	4	4	4	3	**3.6**
P2	2	2.5	2.5	n/a	n/a	4	4	n/a	n/a	**2.8**
P3	3	2	1	5	3	4	4	3	3.5	**3.1**
P4	n/a	n/a	n/a	n/a	n/a	n/a	n/a	n/a	n/a	**n/a**
P5	4	5	3	5	4	5	4	1	4.5	**3.9**
P6	3	4	n/a	n/a	n/a	n/a	n/a	n/a	3	**3.3**
P7	1	3	2	4	n/a	4	3	n/a	n/a	**2.8**
P8	2	3	2	4	2.5	n/a	n/a	3.5	2	**2.7**
P9	3	4	2	5	n/a	4	4	3	3.5	**3.5**
P10	3	5	3	5	n/a	1	1	3	4	**3.4**
P11	4	4	2	4	n/a	4	3	3	4	**3.6**
Mean (SD)	**2.9 (0.9)**	**3.4 (1.2)**	**2.3 (0.7)**	**4.6 (0.5)**	**3.4 (0.6)**	**3.8 (1.1)**	**3.4 (1.0)**	**2.9 (0.9)**	**3.4 (0.7)**	**3.3 (0.4)**

^a^Not used in total and mean calculations. Breakdown of the QUEST 2.0 Likert-scale questionnaire: 1- not satisfied at all, 2- not very satisfied, 3- more or less satisfied, 4- quite satisfied, 5- very satisfied

### Extension and range of motion – unassisted

The researcher was able to fully straighten the index finger MCP, PIP and DIP joints for all but three of the 11 participants, whose muscle tone firmly resisted end-range PIP joint extension. The participants’ fingers were able to bend fully with the researcher’s assistance, so the fingertip touched the palm near the MCP joint. Three participants could not produce visible voluntary motion. Seven additional participants could not produce greater than 30^o^ of active ROM. The participant with the largest active ROM (P9) also demonstrated thumb control. Using the finger goniometer to measure index finger joint angles was generally straightforward, since the fingers were stationary during measurement. For P1–8 and P10, their joint angles at active extension were the same as their joint angles at rest. The participants with active flexion (P4–11) or extension (P9) could hold the flexed or extended position for at least 10 s (i.e. long enough to measure the MCP, PIP and DIP joint angles).

### Extension and range of motion – robot-assisted

The HERO Grip Glove was effective in moving the stroke survivors’ hands, producing statistically significant increases in finger extension and ROM. Each participant showed greater index finger extension at the MCP, PIP and DIP joints (nearer to 0^o^) with robot assistance than without robot assistance (increase of 147^o^, Standard Deviation (SD) 44, *p* < 0.01). A similar increase in middle, ring and little finger extension and middle finger flexion was visually observed for each stroke survivor except P3. Increases in thumb extension and abduction were visually observed for each stroke survivor. The actuators fully contracted and extended for each participant except P3. Due to P3’s tone, the middle, ring and little finger extension cable ties were detached in order for the actuator to fully extend. The robot’s assistance did not fully straighten three participants’ index fingers (P3, 5, 10). Finger tone seemed to reduce as the study progressed, potentially because the participants were more relaxed and the glove moved the hand repeatedly.

The HERO Grip Glove’s assistance restored a large portion of the stroke survivors’ available ROM (69% increase, SD 13, *p* < 0.01), as measured using the Percent of Motion Restored (%MR) metric proposed in [14]. For each participant, the R-A ROM was larger than the active (unassisted) ROM (increase of 145^o^, SD 36, p < 0.01). The R-A flexion joint angles were particularly lower than the passive flexion joint angles.

Three of the four participants with pinch strength generated greater joint flexion when asked to flex their hand to supplement the glove’s flexion assistance, while one participant could not maintain an active grip long enough to measure the change in flexion (P5: Not measured, P7: 15^o^, P9: 60^o^, P10: 40^o^). P1 and P2 did not have pinch strength and did not show additional flexion.

### Grip and pinch strength *–* unassisted

Eight of the 10 participants assessed generated grip force (30.8 N average, SD 30.5). Pinch force was generated by 9 of the 11 participants tested (16.6 N average, SD 11.1). Each participant except P8 needed to use a lateral pinch because their fingers could not be maneuvered to create a tripod pinch. P1 and P2 had flaccid paralyzed hands that could not produce grip or pinch force and P3 could not apply grip force.

### Grip and pinch strength – robot-assisted

The HERO Grip Glove restored grip and pinch strength to the three participants with no grip or pinch strength. The glove provided an average of 12.7 N (SD 8.9) of grip force and 11.0 N (SD 4.8) of pinch force to the participants’ relaxed hands. The participants’ thumbs were not always inserted fully, and this caused much of the variability between participants.

All four participants with pinch strength generated greater pinch forces when they were asked to flex their hand to supplement the glove’s pinch force (P5: 22.2 N, P7: 13.3 N, P9: 8.9 N, P10: 22.2 N). On average, the participants generated greater forces by flexing their hand than by relying on the glove to generate force through their relaxed hand.

### Box & block test performance *–* unassisted

Three of the 11 participants were able to grasp and transfer blocks without arm or hand assistance, using lateral (P9) and tripod (P7, P11) pinch grasps. P4 was able to grasp and transfer blocks with arm support. The other participants were not able to grasp a block. The participants transferred an average of 2.8 (SD 5.3) blocks without the glove.

### Box & block test performance – robot-assisted

All 11 participants were able to grasp and transfer blocks with the HERO Grip Glove. Four participants (P7, 8, 9, 11) did not require any other assistance, six participants (P2, 3, 4, 5, 6, 10) used their unaffected hand to support their forearm and one participant (P1) opted for the researcher to support the forearm due to general fatigue. The participants transferred an average of 2.9 (SD 0.9) blocks each in the button mode and 3.3 (SD 0.5) blocks in the automatic mode. A tripod pinch grasp was used for each grasp.

In the button mode, the 11 participants transferred a combined 32 blocks and failed to grasp the block on 5 attempts. In the automatic mode, the 9 participants assessed transferred a combined 30 blocks and failed to grasp the block on 2 attempts. Grasp assistance was triggered too early on 3 occasions and no blocks were released too early (90% intent detection accuracy). The automatic mode eliminated the need to push a button, saving each participant a few seconds per grasp and the inconvenience of reaching for the button. Each participant mastered the manual mode within 1 min and the automatic mode within 5 min. Each block that was grasped was transferred and released appropriately using both the manual and automatic modes. While using the glove, the most difficult parts of this task were isolating one block from the others and positioning and orienting the hand around the blocks. The robot’s actuation speed limited the number of blocks that could be transferred; however, the participants were content with the robot’s speed because they prioritized a successful grasp and the slower speed allowed them to position their hand more accurately.

### Water bottle task performance – unassisted

None of the 10 participants assessed could complete the water bottle ADL task without assisting their grasp with their unaffected hand. One participant (P1) could not complete the task because they did not have enough grip strength to hold the water bottle. Nine participants could not extend their fingers around the water bottle and required their unaffected hand to push and twist the water bottle into their toned hand. Six participants (P5, 6, 7, 8, 9, 11) were then able to lift the water bottle and remove the lid without arm support and the other participants (P2, 3, 10) required arm support.

### Water bottle task performance – robot-assisted

With the HERO Grip Glove, each participant completed the water bottle ADL task (i.e. grasp bottle, remove lid, lift bottle, lower bottle, attach lid, release bottle). Seven participants (P1, 2, 5, 6, 8, 10, 11) did not require any support from their unaffected hand during grasping. The other four participants (P3, 4, 7, 9) stabilized the water bottle to keep it from tipping or being pushed out of the glove while closing. Six participants (P3, 6, 7, 8, 9, 11) lifted the water bottle and removed the lid without arm support and the other participants (P1, 2, 4, 5, 10) required arm support. The participants were trained in stages using a hand-over-hand technique for up to 3 min and were assessed using the HERO Grip Glove’s manual mode.

### Fork task and pen task performance – unassisted

Two of the seven participants assessed (P7, P9) were able to grasp the fork from the table and lift and manipulate it without assistance. However, they could only grasp the fork with a lateral pinch grasp and were not able to orient the fork appropriately for stabilizing food. P8 stretched his fingers into extension with the other hand, grasped the fork loosely and dropped it while lifting. Four participants (P1, 2, 6, 10) could not pick up the fork from the table or grasp the fork when placed in their hand. The average score on the Fork Task was 3.0 (SD 2.5) using the TRI-HFT scale.

### Fork task and pen task performance – robot-assisted

With the HERO Grip Glove, all eight participants assessed were able to grasp the fork using a tripod pinch and lift and manipulate the fork, giving an average score of 6.0 (SD 0) using the TRI-HFT scale. The grasp orientation was appropriate for stabilizing and eating food. Two participants (P3, P8) grasped the fork from the table without assistance and P8 could also lift and manipulate the fork without assistance. Six participants used the unaffected hand to place the fork in the affected hand in order to firmly grasp the fork in the correct orientation for eating (P1, 2, 6, 7, 9, 10). Only the participants that required arm support without the glove required arm support with the glove (P1, 2, 3, 10). The participants did not reach normal function because they did not show the speed, consistency, in-hand manipulation or force of an unaffected hand. Each participant was able to release the fork with the glove’s assistance.

Three participants (P2, 6, 10) that were unable to grasp the fork and whose dominant hands were affected by the stroke attempted to write with a pen while wearing the HERO Grip Glove. Each participant used the unaffected hand to position the pen in the affected hand and was able to firmly grasp the pen in a tripod pinch grasp. P2 and P10 were able to write with arm support from the other hand. P6 was able to write without arm support, but arm support helped to reduce shoulder abduction. The pen would inconveniently slip or rotate when heavy pressure was applied (~4lbs) so a marker was used in repeat trials for P10. The participants’ writing was not neat but was legible.

### User satisfaction with the HERO Grip Glove

The stroke participants completed the QUEST 2.0 questionnaire to provide feedback on how satisfied they were with the HERO Grip Glove and its ability to meet their hand mobility needs throughout their daily routines. The Likert-scale rankings are shown in Table 6 (from 1 “not satisfied” to 5 “very satisfied”). The participants were “more or less satisfied” with the glove, giving an average score of 3.3 (SD 0.4). The overall average scores did not vary greatly between participants (2.7 to 3.9). Safety and security was given the highest rating (4.6) because the glove did not produce pain and did not extend the fingers too far or too fast. Ease of donning was given the lowest rating (2.3). A single assistant was required to position the fingers, and especially the thumb, into the glove. The HERO Grip Glove required, on average, 180 s to don (SD 55) and 23 s to remove (SD 10). The final four participants in the study, chronologically, were asked to doff the glove independently and did so in less than 30 s. A mixture of satisfied and unsatisfied reviews was given for each of the other sections. There are no correlations evident between the QUEST rating and the quantitative measures of stroke severity, ROM, grip and pinch strength or ADL task performance with or without the glove.

The participants provided detailed technical observations and suggested pragmatic and insightful solutions for improving the HERO Grip Glove. Five participants (P1, 3, 5, 8, 11) noted that they would prefer an automatic mode over the manual mode, but that the current automatic mode needs improvement because it was both difficult to trigger and triggered too often during daily tasks like using a fork or water bottle. Four participants (P3, 5, 9, 11) requested that the glove provide greater grip strength, while P1 was satisfied with the grip strength. Four participants (P2, 7, 10, 11) requested the glove’s fingers, and especially the thumb, fit more snugly. The glove caused minor discomfort on P11’s long fingernails and P5 and P9’s thumb tip where the tendon was anchored. Three participants (P1, 7, 11) commented that the glove was not heavy on its own, but that the affected arm itself was “heavy” or difficult to move. Additionally, P2 and P8 requested the battery pack be moved proximally to be hidden under a sleeve, P7 liked the wrist brace’s comfort and stability, P9 requested a more pliable wrist brace and the ability to don the glove independently. At least five stroke participants requested follow-up sessions to use the glove and incorporate it into a therapy program without being prompted.

## Discussion

A third of stroke survivors do not recover the hand function required to use their affected hand in daily tasks, leading to dependence in ADL and further declines in hand and arm function [3]. Wearable hand robots have the potential to restore stroke survivors’ range of motion and grip strength, which may enable them to have greater independence and mitigate their declines in function from disuse [40]. The HERO Grip Glove’s design was motivated by the lessons learned while evaluating previous wearable and untethered hand robots [14, 15].

The HERO Grip Glove incorporates the following novel design features:

- A single-actuator system that assists five-finger extension and thumb abduction.
- A single-actuator tripod pinch grasp system that assists index and middle finger flexion and thumb opposition and conforms to various object geometries.
- A wearable hand robot that is lightweight, untethered, fully contained on the hand and forearm and incorporates a fold-over wrist brace and open-palm glove for wrist support and quick donning.
- A wearable hand robot that is affordable and available through open-source manufacturing for stroke survivors to use throughout their daily routines.

The assistive capabilities and usability of this novel wearable hand robot were evaluated with the largest number of stroke survivors with severe hand impairment to date. Key findings of this study were:

- The HERO Grip Glove provided the finger extension and grip force required for stroke survivors to stabilize water bottles, wooden blocks, forks and pens.
- The HERO Grip Glove produced statistically significant improvements in finger extension (147^o^, SD 44), range of motion (145^o^, SD 36) and ADL performance with large objects (increase of 2.3 out of 7, SD 1.2) and small objects (increase of 3.0 out of 7, SD 2.5) and provides grip (12.7 N, SD 8.9) and pinch force (11.0 N, SD 4.8).
- The stroke survivors were more or less satisfied with the HERO Grip Glove’s design and usefulness for their daily routines (3.3 out of 5, SD 0.4) and provided suggestions on which specifications should be modified to increase satisfaction.

The HERO Grip Glove is ready to be trialed by stroke survivors with a Chedoke McMaster Stage of Hand less than five to enable greater use of the affected upper extremity while performing daily tasks in therapy clinics and at home.

### Enhancing finger extension and range of motion

The HERO Grip Glove enhances index finger extension (by 147^o^) and ROM (by 145^o^) to a greater extent than previous designs. Key contributors to the performance increases were mounting the 80 N actuators to a wrist brace and adding flexion tendons on the palmar side. Although integrating a wrist brace was negatively weighted in a previous hand robot metric [21], wrist braces have been used to provide a rigid mounting point for actuators and to apply strong forces without orthosis migration [15, 17, 41, 42]. In addition to these features, the wrist brace provided us with an anchoring point for the palmar tendons, which was necessary to create the opposition required for a tripod pinch instead of a lateral pinch. The participants and therapists involved in our study preferred the wrist brace because it felt comfortable, protected their tendons from hyperextending, and kept the wrist in approximately 30^o^ of extension, which is typical for grasping and may have reduced tone to ease finger extension. Using the glove to repeatedly stretch the fingers may also reduce tone and enable the glove to further extend the fingers.

### Strengthening grip force assistance

The majority of participants in this study produced large grip and pinch forces without robot assistance, in comparison to previous studies [12, 14]. Although these participants could activate their muscles to supplement the glove’s grip force, they desired greater grip force from the glove. The participants mentioned that it was challenging to generate a controlled grip force without assistance and that maintaining grip force while moving the arm was highly fatiguing and increased spastic responses. The HERO Grip Glove generated greater grip and pinch forces (12.7 N and 11.0 N on average) than many previous devices [12–14, 16, 43] and these forces enabled participants to grasp objects that they could not grasp otherwise. However, larger grip forces should be strived for if these forces do not sacrifice other usability criteria, since these grip forces are less than age-matched norms of 294-542 N [44] and certain everyday tasks and sports activities, such as closing zippers, inserting a fork into dense food, writing with a standard pen and holding a tennis racquet, golf club or fishing rod, can require greater than 15 N of grip and pinch force [10, 45]. In addition, standardized equipment and protocols are needed to evaluate the force, pressure and kinematic outputs of soft hand robots on the finger joints and skin before definitive comparisons can be made (e.g. using fine resolution digital dynamometers and anatomical testing apparatus [46, 47]).

Three solutions for increasing the glove’s grip force are to further pretension the grip tendons, increase the number of grip tendons as in [10, 48] and create custom-fitting gloves that keep the thumb from migrating. Finger extension was prioritized over grip force during the cable tie adjustment period in this study for consistency; however, if grip force, ADL performance or individual requests were prioritized during cable adjustment this may have resulted in greater satisfaction. If the gloves were customized to each participant, fitted gloves would be created and grip tendons would be added to the ring and little finger for users with non-clenched hands as these features would not largely affect donning time but would increase grip stability and force. For participants that can generate unassisted grip or pinch force, the glove may only need to provide a portion of the required grip force [49] or assist the user in generating an efficient grip posture or controlled force to complete the task safely while reducing spasticity and fatigue.

Assessing participants’ performance on all components of the CAHAI assessment, as in [41], would be a useful next step for determining which tasks the glove’s assistive capabilities are most beneficial for. With further training sessions, the participants could learn how to best activate their muscles to support the glove’s motion and force to complete the tasks more effectively and promote neuromuscular recovery.

### Usability during activities of daily living

Many bimanual tasks can be compensated for using adaptive equipment (e.g. one-handed rocker knives and button hooks and voice-controlled appliances) and other body parts (e.g. thighs, teeth). Therefore, stroke survivors place high expectations on hand robots to enable their affected hand to exert strong forces and produce multiple grips similarly to their unimpaired hand [50].

Stroke survivors were “more or less satisfied” with the HERO Grip Glove (rated 3.3 out of 5). This provides motivation for the field of robotic hand orthoses considering that lower-limb exoskeletons and home assistance robots are rated in-between 3 and 4 on the QUEST scale and are becoming widely used for assistance and rehabilitation [51, 52]. However, the stroke survivors provided a number of reasons for not being “very or extremely satisfied”, and this motivates key areas for specification modification and device improvement:

• Increase the number of grip tendons to increase grip force.

• Tailor the gloves to fit snugly, prevent orthosis migration, distribute pressure and ease donning.

• Optimize the location of the flexion tendons and actuator to reduce obstructions in the grasp workspace.

• Integrate powered or passive arm supports for weaker arms.

### User preferences for hand robot design features

In previous hand robot usability studies [15, 16], stroke participants requested aesthetic changes, weight reduction and waterproofing. In our study, the participants frequently noted that aesthetics were not a high priority for use inside the home or clinic and that the glove’s effectiveness in enabling them to perform daily tasks independently was their highest priority. The glove did not have any noticeable effect on the participants’ ability to move the arm during the tasks. The participants were pleased that the HERO Grip Glove was quiet and felt safe and that being untethered would allow the glove to be used more conveniently throughout their daily routines.

The participants voiced their preference for controlling the assistance without their unaffected hand and easily learned to use the automatic mode for one specific task. However, this trial and previous trials have shown that for severely affected arms a well-placed button is more usable over a variety of tasks than motion triggered control and often users’ electromyography signals are too weak or sporadic to be used for control [17, 53, 54]. Most participants required practice to learn how a fork or pen should be oriented in the affected hand and suspected they would need similar practice in their household before understanding how to best incorporate the gloved hand into their daily routine.

### Use cases for hand robots after stroke

The participants were interested in using the glove for stretching and therapy sessions and to monitor changes in tone, function and ADL performance over time. Participants have been shown to activate their forearm muscles to supplement the glove’s grip force during their ADLs [54], which may promote upper extremity neuromuscular recovery through continued use. Integrating the HERO Grip Glove into therapy programs may provide additional neuromuscular recovery, as previous robotic gloves have been integrated into therapy exercises and have provided neuromuscular recovery of 3.3% of the total achievable recovery, using the Fugl-Meyer Upper Extremity assessment [18, 41]. Integrating the HERO Grip Glove into therapy exercises and daily routines is an important next step for investigating if larger improvements in neuromuscular recovery can be reached.

### Personalization and accessibility of robotic gloves

A one-size-fits-all solution does not seem optimal for the severe stroke population. For stroke survivors with functional arms, flaccid hands and few contraindications, a HERO Grip Glove is likely a standalone device that will enable them to perform more daily tasks independently after an initial training session to practice donning and operating the glove independently and incorporating the affected hand into desired tasks. Stroke survivors with clenched hands will require assistance to don the glove and using fewer flexor tendons will further aid donning. Those with weak, flaccid or spastic arms will need arm supports and additional training to realize the ADL benefits of an active hand. Stroke survivors with the ability to flex and extend their hands are not likely to find this glove useful unless much stronger grip strength or individual finger control is added. Further evidence on how the device specifications impact performance for specific subgroups (e.g. high tone, flaccid hand and arm) is required to validate our interpretations and direct therapists and users in selecting between personalization options.

The HERO Grip Glove has many features that can be customized for each user. The cable ties can be adjusted to provide greater finger extension or greater grip force, the number and location of grip tendons can be modified, the glove and wrist brace can be individually sized and the IMU control thresholds can be adjusted based on the user’s arm mobility. Often such customizations keep these devices out of the affordable range for stroke survivors on limited budgets or without insurance coverage. Since the HERO Grip Glove has a low component cost ($300) and can be manufactured with common hand tools in under 5 h, it is a good fit for being produced by volunteers specifically for each intended user to meet their performance and affordability needs. For this reason, the HERO Grip Glove hardware and software designs are open-source and available at https://www.makersmakingchange.com/project/hero-glove/. This method also allows for a flow of conversation between the users, volunteers and designers to improve the glove’s usefulness, ease of use, reliability and instruction manuals based on real-world experiences.

### Limitations in the study design

A limitation of this study is that the HERO Grip Glove was not trialed with the same participants as the HERO Glove, since there was not enough time during the two-hour study session to repeat the assessments three times (i.e. baseline (No Glove), HERO Glove, HERO Grip Glove). The HERO Grip Glove was tested against the baseline condition, as this would be a more attainable study for other researchers to replicate using their own hand robot designs. Comparative studies between hand robots should be completed in the future to determine the how people after stroke would select between design tradeoffs, such as grip assistance versus fewer actuators, assistive capability versus ease of donning, integrated devices versus devices that store robot components on the hip or back [16, 20, 43]. A second limitation is that arm support was provided by the participant or researcher if needed, which is difficult to standardize between studies.

## Conclusions

This study evaluated the assistive capabilities and usability of the HERO Grip Glove. The qualitative and quantitative data collected provides guidance for future wearable hand robots and feedback from people after stroke on the modifications and features they desire. For stroke survivors with severe hand impairments, the HERO Grip Glove’s assistance improved finger extension, range of motion, grip strength for those without active grip strength, and performance on components of daily living tasks. The stroke survivors were more or less satisfied with the HERO Grip Glove. The stroke survivors suggested design improvements, such as assisting arm motion, providing greater grip strength assistance, tailoring the gloves individually to fit each user, and designing the gloves to be easier to don independently. In future clinical research the HERO Grip Glove will be distributed amongst a variety for populations with grip and range of motion hand impairments following stroke, spinal cord injury, muscular dystrophy and cerebral palsy. The HERO Grip Glove’s assistive capabilities and usability will be evaluated in therapy clinics, users’ homes and in outdoor environments using tasks that are selected as meaningful by the device users.

## Supplementary information

**Additional file 1 : Figure S1.** Pictorial representations of the index finger extension and range of motion (rom) results in Table 3.

## Data Availability

The datasets used during the current study are available from the corresponding author upon reasonable request.

## Abbreviations

%MR: Percent of Motion Restored
ADLs: Activities of Daily Living
BBT: Box and Block Test
CAHAI: Chedoke Arm and Hand Activity Inventory
CMSA: Chedoke McMaster Stroke Assessment
DIP: Distal Interphalangeal
FMA-S: Fugl-Meyer Assessment – Sensation
HERO: Hand Extension Robot Orthosis
IMU: Inertial Measurement Unit
MCP: Metacarpophalangeal
MMAS: Modified Modified Ashworth Scale
MTS: Modified Tardieu Scale
P: Participant
PIP: Proximal Interphalangeal
QUEST: Quebec User Evaluation of Satisfaction with Assistive Technology
R-A: Robot-Assisted
ROM: Range of Motion
SD: Standard Deviation
TRI-HFT: Toronto Rehabilitation Institute – Hand Function Test
