# Supplementary material for: Hand Extension Robot Orthosis (HERO) Grip Glove: enabling independence amongst persons with severe hand impairments after stroke
Source: J Neuroeng Rehabil. 2020 Feb 26;17:33. doi: 10.1186/s12984-020-00659-5 (PMC7045638; doi:10.1186/s12984-020-00659-5)
Supplement: Supplementary file 1 — Additional file 1 : Figure S1. Pictorial representations of the index finger extension and range of motion (rom) results in Table 3. [file 12984_2020_659_MOESM1_ESM.docx]

Supplementary Figure I. Pictorial Representations of the Index Finger Extension and Range of Motion (ROM) Results in Table III

| 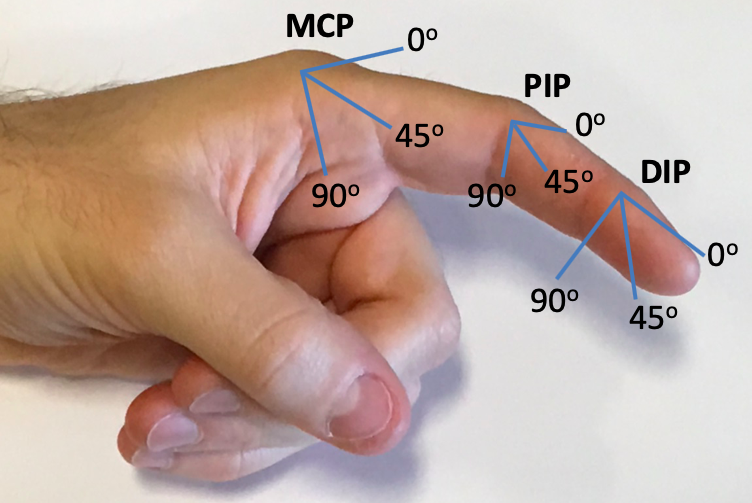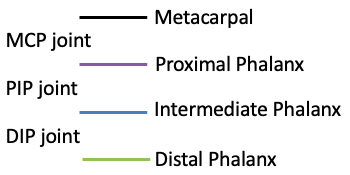     \| P.Ex \| Passive Extension \| \| --- \| --- \| \| P.Fl \| Passive Flexion \| \| A.Ex \| Active Extension \| \| A.Fl \| Active Flexion \| \| R.Ex \| Robot-Assisted Extension \| \| R.Fl \| Robot-Assisted Flexion \| | **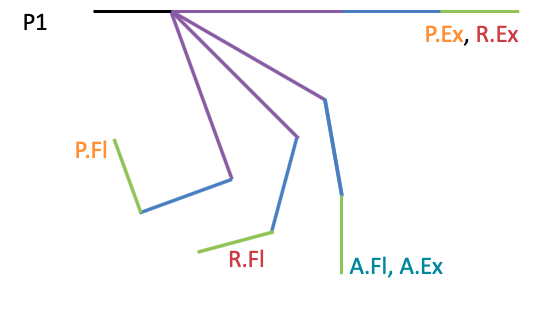** |
| --- | --- | --- | --- | --- | --- | --- | --- | --- | --- | --- | --- | --- | --- |
| **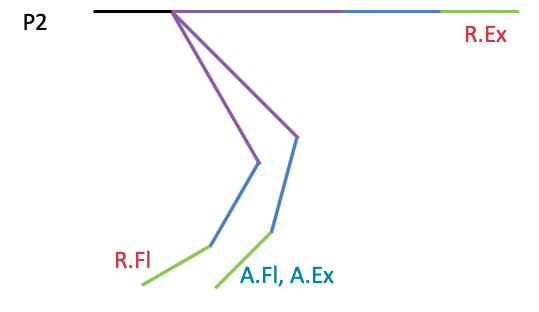** | **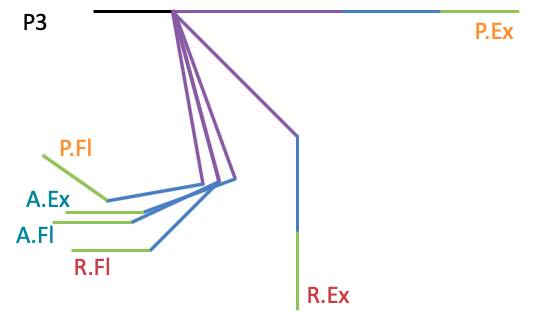** |
| **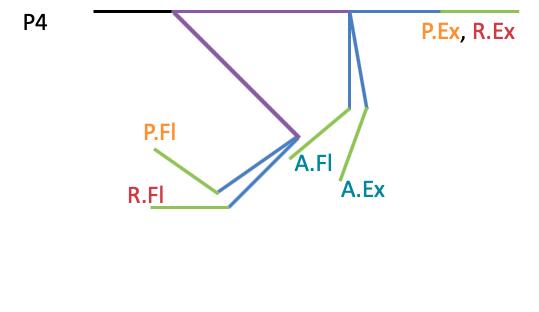** | **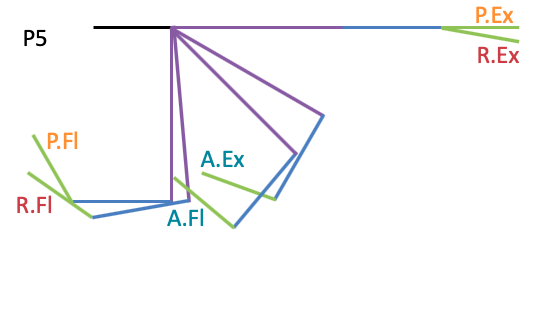** |
| **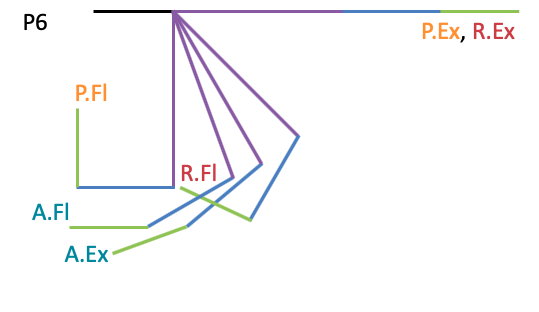** | **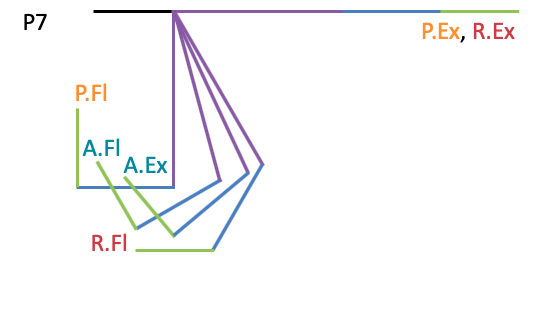** |
| **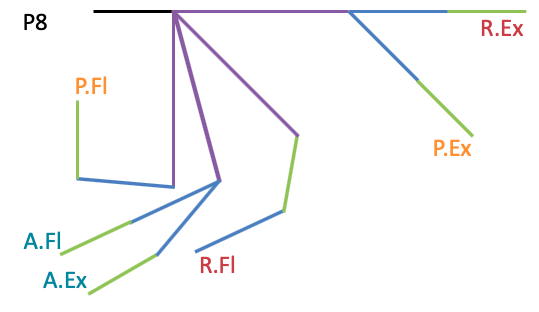** | **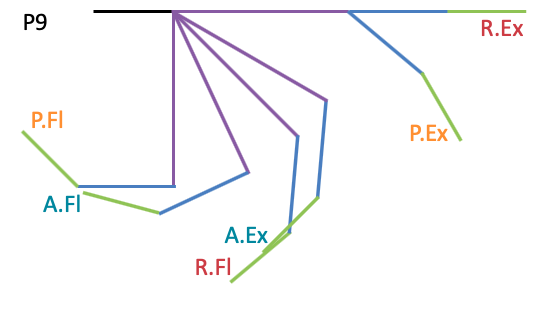** |
| **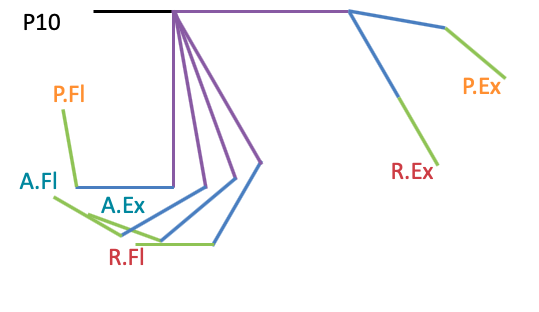** | **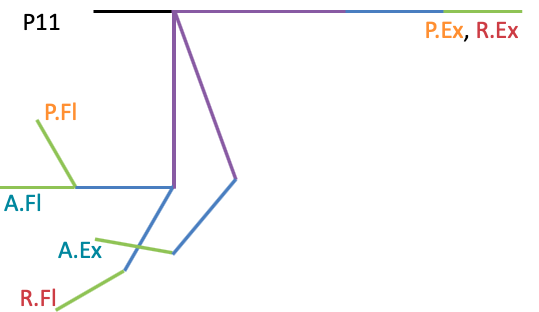** |

These pictorial representations of index finger joint angles for P1 through P11 assume that the ratio between proximal, intermediate and distal phalanx lengths (e.g. 45mm, 25mm and 20mm lengths, respectively) are constant across participants.
